# Simulation of Melting Efficiency in Laser Cutting of Hardox 400 Steel

**DOI:** 10.3390/ma15207192

**Published:** 2022-10-15

**Authors:** Constantin Cristinel Girdu, Catalin Gheorghe

**Affiliations:** 1 Department of Manufacturing Engineering, Transilvania University of Brasov, Eroilor Street 29, 500036 Brasov, Romania; 2 Department of Engineering and Industrial Management, Transilvania University of Brasov, Eroilor Street 29, 500036 Brasov, Romania

**Keywords:** melting efficiency, analysis of variance, response surface method, HARDOX 400, laser CO_2_, melted volume

## Abstract

Laser cutting has experienced a sharp development in recent years due to the advantages it implies in industrial production, the most important being: great diversity of processed materials, reduced cutting time, low processing cost, small percentage of removed material, and low impact on the natural environment. The problem of energy has become acute in the last year, so a new direction of research has taken shape, consisting of the optimization of the high energy consumptions involved in laser cutting. The objective of this research is to develop a computational and experimental model to estimate the melting efficiency. Additionally, the research seeks to establish some mathematical relationships that describe the law of variation of the melting efficiency depending on the input parameters in the CO_2_ laser cutting. The experimental determinations were carried out on Hardox 400 steel plates of 8 mm thickness. The input parameters were laser power, assist gas pressure, and cutting speed. The experimental data were statistically processed, and the results were verified with the Lagrange interpolation method. It was found that the maximum melting efficiency is influenced mainly by laser power (F = 3.06; *p* = 0.049), followed by speed and pressure. The results obtained show that the melting efficiency varies in the range (13.6–20.68) mm^3^/KJ. The maximum value of the melting efficiency (20 J/mm^3^) was obtained when the laser power was 5100 W, the cutting speed 1900 mm/min, and the gas pressure 0.5 bar, and the minimum efficiency under conditions of speed setting at 1700 mm/min and laser power of 5000 W. Linear and quadratic regression models were established to estimate the global mean efficiency according to two independent variables that act at the same time. The established calculation relationships contribute to the improvement of the literature and constitute a tool for practical applications. The results obtained allow the modeling of cutting parameters and the optimization of production costs in industrial processes that use laser cutting.

## 1. Introduction

Among the challenges that economies have faced in the last year is the issue of energy. The increase in electricity, heat, and fuel prices caused a chain reaction in the national economies, contributing to the dynamics of inflation, the reduction of consumption, and the decrease in the volume of the company’s activity. The manufacturing industry is characterized by high material and energy consumption. To face these trends, the manufacturing industry has focused on reducing raw material consumption, lowering energy and fuel consumption, increasing the degree of energy independence, using green technologies, reducing losses and the resulting waste, and developing renewable energy sources.

One of the procedures frequently encountered in the industrial environment is cutting. The process can be carried out with conventional or nonconventional technologies. One of the unconventional technologies frequently used in the manufacturing industry is laser cutting. Such a process consists of using laser radiation to cut metallic or non-metallic materials. The technology presents numerous advantages such as: complex surfaces obtained in a short time, cutting precision, and lack of mechanical cutting forces, narrow zone affected by heat, reduced cutting width, no tool wear, and small amount of waste generated. In addition, it is considered a method friendly to the natural environment; therefore, it has established itself as a sustainable alternative to traditional processing methods [1,2,3]. As a processing method, it is found in several industries such as: metallurgy, equipment manufacturing, machinery and machines, road transport vehicles manufacturing, and others [4,5]. Currently, laser cutting is frequently used, being one of the modern processes for cutting a wide variety of materials. Research was developed on laser cutting of metallic materials such as: mild steels, alloy steels, aluminum, nickel alloys, titanium, or very hard materials [3,6,7,8].

Hardox 400 steel was chosen for conducting the experimental research. The arguments that led to the choice of material were the following: the large number of industrial applications; the special mechanical, physical, and chemical properties; and the small number of scientific articles related to this steel. The main characteristics of Hardox 400 steel are: high wear resistance, excellent corrosion resistance, good weldability, good strength-to-weight ratio, good cold plastic deformation properties, high fatigue resistance, and good impact load resistance. In addition, the material is not intended for additional heat treatment [7,9].

Mechanical and thermal characteristics have led to the use of steel in the industries listed above, in the case of applications that require resistance to abrasion with impact and good cold bending properties [5,6,7,8]. The cutting of Hardox 400 steel using conventional technologies leads to high wear of the cutting tools under conditions of average roughness values of the cut surfaces [8,9,10].

Laser processing of Hardox 400 is, however, hard due to the physical and chemical properties of this steel. As it exhibits high thermal conductivity, high melt viscosity, and high absorptivity, laser cutting is an alternative process that can ensure efficient steel processing. Such properties constitute an additional argument to choose the material as an object of research [10,11,12,13].

Melting efficiency is closely related to energy consumption. This plays a decisive role in fiber or CO_2_ laser cutting. Having such a benchmark, the authors determined other important sizes in laser cutting, such as linear energy and cutting efficiency. Such sizes were the subject of other published articles. For the continuation of these investigations, the authors propose the concept called melting efficiency, which means the volume of material melted with 1 Joule energy consumption. The calculation relationship is as follows:

(1) $$E_{m}=\frac{V}{E}[\frac{{\mathrm{mm}}^{3}}{J}],$$

where V is the volume of the molten material (mm^3^), and E is the laser energy used to melt the steel (J). If the laser irradiation time is considered, the relation (1) becomes:

(2) $$\frac{E_{m}}{t_{i}}=\frac{V}{E\cdot t_{i}}=\frac{Q_{f}}{E}[\frac{{\mathrm{mm}}^{3}}{J\cdot s}],$$

where Q_f_ is the melt flow rate (mm^3^/s), t_i_ is laser irradiation time (s).

The mass melted by consuming the laser energy E_m_ is given by the relation:

(3) $$∆{m=E}_{m}\cdot \rho =\frac{m}{E}\;[\frac{g}{J}],$$

where ∆m is the molten mass (g), ρ is the material density (g/mm^3^). These relationships are defined as energy indicators for melting.

When laser radiation interacts with the material, melting occurs. The laser spot possesses a large energy amount that acts on the material, generating a liquid mass of material that moves. The process is based on energy transfer, absorbency, and strong heating in a very short time. The superheated melt is removed by the pressurized gas jet. Because the melting process in the material is hydrodynamic, we will determine the equation of melt motion in a liquid state considered spherical. The forces acting on the melt are droplet gravity (G), gas resistance force (F_g_), and Stokes force (F_s_) between the droplet and the side surfaces. The simultaneous action of the forces determines the following equation of motion:

(4) $${G\;-\;F}_{g}-\;2\cdot F_{s}=m\cdot a,$$

(5) $$m\cdot g\;-\;k\cdot v\;-\;6\cdot \pi \cdot \rho \cdot r\cdot v\;-\;6\cdot \pi \cdot \rho \cdot r\cdot v=m\cdot \frac{\mathrm{dv}}{\mathrm{dt}},$$

(6) $$m\cdot g\;-\;k\cdot v\;-\;6\cdot \pi \cdot \rho \cdot D\cdot v=m\cdot \frac{\mathrm{dv}}{\mathrm{dt}},$$

where m is melt mass (g), g is gravitational acceleration (m/s^2^), a is acceleration (m/s^2^), k is resistance coefficient (kg∙s), ρ is viscosity (N∙s/m^2^), r is melting radius (mm), dt is infinitesimal time (s), v is melting speed (m/s) and D is diameter of melting (mm).

From Equation (6), the following form results:

(7) $$\mathrm{dt}=m\cdot \frac{\mathrm{dv}}{m\cdot g\;-\;k\cdot v\;-\;6\cdot \pi \cdot \rho \cdot D\cdot v}.$$

The following expression is denoted by u:

(8) u=m·g - k·v - 6·π·ρ·D·v.

By differentiation, we obtain:

(9) $$\mathrm{du}=(-\frac{k}{m}\;-\;\frac{6\cdot \pi \cdot \rho \cdot D}{m})\cdot \mathrm{dv}.$$

By a change of variable, it results in:

(10) $$\mathrm{du}=(-\frac{k}{m}-\frac{6\cdot \pi \cdot \rho \cdot D}{m})\cdot \mathrm{dv}.$$

Through integration, the following relationship results:

(11) $$t\cdot (-\frac{k}{m}-\frac{6\cdot \pi \cdot \rho \cdot D}{m})=\mathrm{lnu},$$

(12) $$t\cdot (-\frac{k}{m}-\frac{6\cdot \pi \cdot \rho \cdot D}{m})=\ln(m\cdot g\;-\;k\cdot v\;-\;6\cdot \pi \cdot \rho \cdot D\cdot v).$$

From relation (12), it follows the speed of melting:

(13) $$v=\frac{m}{k+6\cdot \pi \cdot \rho \cdot D}\cdot {(g\;-\;e}^{-\frac{k}{m}\cdot t\;-\;\frac{6\cdot \pi \cdot \rho \cdot D}{m}\cdot t)}.$$

Through the elementary displacement of the melt, an infinitesimal variation of heat takes place, which causes the temperature to rise by dT.

(14) $${\mathrm{dQ}=\mathrm{dL}}_{r}$$

(15) $$m\cdot c\cdot {\mathrm{dT}=-F}_{r}\cdot \mathrm{dr},$$

(16) $$m\cdot c\cdot \mathrm{dT}=(k+6\cdot \pi \cdot \rho \cdot D)\cdot v^{2}\cdot \mathrm{dt},$$

where dQ is the infinitesimal variation of heat, dL_r_ is the infinitesimal variation of the resistant mechanical work, c is specific heat (J/g∙K), F_r_ is resistance force, T is melting temperature (K). By integration, results:

(17) $$m\cdot c\cdot T=(k+6\cdot \pi \cdot \rho \cdot D)\cdot v^{2}\cdot t.$$

Taking into account the value of the speed determined previously, the value of the melting temperature can be estimated. In the calculations, the term f = (k + 6∙π∙ρ∙D) is dropped as it has no significant influence. This results in a formula that determines the value of the temperature in the melt (T):

(18) $$T=\frac{v^{2}\cdot t\;}{m\cdot c}\cdot f=1730\;(K),$$

where t is the duration of the movement. The result is close to the existing values in the literature [14,15]. The speed value is consistent with the result obtained for the drop in molten material set by Samarjy and Kaplan [16]. Using the data in Table 1, we obtain the speed of melting through the material and its temperature.

Speed and temperature are two important physical quantities that describe the behavior of the melt through the material. The first is a dynamic size, and the second is a state size. With their help, we can estimate the kinetic and internal energy of the melt that contribute to the laser melting and cutting process.

## 2. Synthesis of the Literature

### 2.1. Synthesis of Literature Related to Hardox Steel

The review of the literature allowed us to establish the current state of research on CO_2_ laser cutting of steels. In the first part of this section, research that studied Hardox steel and their orientation was identified. One such piece of research was conducted by Naik and Maity (2020). The authors chose Hardox 400 steel as an experimental material in the form of 10 mm thickness plates. In a laser plasma cutting installation, the authors analyzed the influence of several gases (air, oxygen, argon, and nitrogen) on the material [17]. Ramos et al. studied the extent to which the surface condition of the plates before cutting influences the quality of the machined parts. The authors used Ruukki and Hardox steel plates with thicknesses of 6, 10, and 15 mm. The cutting was performed with a CO_2_ laser installation. The results showed certain quality differences between the steels studied [12]. Dahil et al. compared four processes to cut Hardox 500 steel plates. The processes were plasma cutting, laser cutting, wire erosion, and abrasive water jet cutting. All were analyzed from the point of view of the microstructure and hardness of the obtained surfaces [13]. A similar orientation was identified by Szataniak et al. The authors followed the results of cutting technologies (oxygen, laser, plasma, and water jet cutting) on the hardness of Hardox 400 and 450 steels. To choose the cutting process, the authors recommended considering the thickness of the material, its characteristics, and the cutting precision [18]. Hlavac et al. analyzed the result of the abrasive jet speed on Hardox 400 steel. The authors used five different speeds, measured surface roughness, and analyzed surface topography. The authors found that the roughness increased with the crossing speed. Another finding of the authors is related to the variation in roughness between the lower and upper edges [19].

Research on Hardox steel was found to be limited. Three representative works that studied the laser cutting of Hardox steel were identified, insufficient from the authors’ point of view, considering the large number of applications in different industries where this material is used.

### 2.2. Synthesis of the Parameters in Laser Cutting

The synthesis of the literature continued with the identification of the works that followed CO_2_ laser cutting of steels, regardless of their class, with the aim of identifying the parameter or combination of parameters followed by various researchers. A first identified work was performed by Parthiban et al., who studied CO_2_ laser cutting of 304 stainless steels. The parameters studied during cutting were speed, power, and gas pressure. Using the Box–Behnken design, the response surface method, and a genetic algorithm, the authors determined the influence of each parameter on the cutting width [20]. Jarosz et al. investigated the effect of speed on surface roughness and heat affected zone (HAZ) in laser cutting. The material used was AISI316L stainless steel [3]. Tahir et al. took a similar approach, using the RSM method and a Box–Behnken design, to investigate the cutting width and the HAZ during the CO_2_ laser cutting of ultra-strength steel 22MnB5 [21]. Miraoui et al. investigated three parameters: speed, laser spot diameter, and power, to determine the thermal effect on the cut surface of steel using CO_2_ laser cutting. The monitored parameters were the depth of the molten zone, the depth of HAZ, and the micro hardness surface [22]. Anghel et al. studied the CO_2_ laser cutting of 304 stainless steels. The output parameter the authors sought was surface roughness. The input parameters were: speed, power, gas pressure, and focal position [23]. Another piece of research focused on CO_2_ laser cutting was realized on different grades of steels by Patidar and Rana (2018). The authors studied the influence of parameters (speed, power, and pressure) and their effects on several overlapping steel plates cut [24]. The same input parameters were studied by Rajesh et al. The output variables were surface roughness and cutting width; the material used was SS-304 stainless steel [25]. The same steel was studied by Kotadiya and Pandya (2016) with the help of the ANOVA and RSM methods. The authors tried to establish the optimal combination of selected parameters (speed, pressure, and power) on the cut surface roughness [26]. In the paper conducted by Patel and Bhavsarb (2021), the influence of several parameters (power, duty cycle, speed, frequency, and pressure) on surface roughness, taper angle, and heat affected zone were analyzed. The material was 10 mm thick hard steel, and the experimental data was processed with the RSM method [6]. Shrivastava and Pandey (2018) presented a multiple regression and an algorithm model to study the deviation, taper, and width of the Inconel 718 steel laser cutting [27].

From the literature on the CO_2_ laser cutting of different steels, it can be seen that most of the research had as its object the determination of the effect of several variables on an output size. The variables that appear with the highest frequency in the research presented above are speed, pressure, and power. Starting from this conclusion, in this article, the three sizes were considered as input variables.

### 2.3. Synthesis of the Literature Related to the Energy Used in Laser Cutting

Research into the literature continued with the identification of works published in journals or volumes with international visibility, oriented in the area of the high energy consumption that laser cutting involves. To grow the efficiency of laser cutting technology, Madić et al. analyzed the impact of input parameters on cutting quality, productivity, costs, energy, and resource efficiency. The authors chose CO_2_ laser fusion as a cutting method. A mathematical model was designed to estimate the process efficiency of AISI 304 stainless steel. The cutting efficiency was defined as the power required to melt the volume per unit time and the laser power used [28]. The cutting width was modeled in a function of speed, focal position, and power. The results obtained showed that the dominant effect on efficiency is determined by the focus position, followed by speed and power [28]. However, we found the incorrect use of productivity instead of yield. Productivity is the fundamental parameter in the area of human resources, being frequently confused with yield because it presents conceptual and computational similarities. Another work oriented toward laser energy was developed by Pocorni et al. The authors considered cutting efficiency to be a fundamental quantity in laser processing. Cutting efficiency was defined by the area created per joule of laser energy. The authors applied it to stainless steel and mild steel depending on the material type, laser wavelength, thickness, and power. The research made contributions regarding the variation of cutting efficiency as a function of material thickness [29]. Guarino et al. performed a technical, economic, and ecological study related to the process of CO_2_ laser cutting compared to selective laser melting. The material chosen was 316L stainless steel. Among the technical results obtained by the authors, differences in roughness and tensile strength attracted attention. Economic profitability is higher for CO_2_ laser processing from the authors’ calculations. Measured environmental impact shows that the laser cutting process is about 2.5 times more environmentally friendly compared to selective melting [30]. Fomin et al. conducted a study of the energy balance in cutting stainless steel sheets. The authors performed a comparative analysis between the fiber laser and the CO_2_ laser. For this purpose, they used two dimensionless energy parameters, the laser power and the Peclet number. The energy per melt unit volume was calculated for all lasers. The authors calculated an energy consumption equal to 26 J/mm^3^ regardless of the type of laser and assisted gas (nitrogen or oxygen) [31]. Khorasani et al. have developed a model to anticipate the absorption by laser layer fusion (LB-PBF) of IN718. The authors made contributions to the morphology of the melt and the melt tracks. The mathematical model designed and tested by the authors contributed to the estimation of an absorption ratio of the heat generated during processing [32]. Recent research was also consulted that had as its objective the melting of the material even if it targeted other technologies such as selective laser melting [33] or fusion additive manufacturing [34].

From the synthesis of research oriented towards the energy consumed during laser cutting, the small number of published works is noteworthy. The orientation of the authors on this subject is different. Even the definition of the concept expressing the energy consumption for cutting the material is different. However, all authors who addressed energy consumption concluded that it is a fundamental size that must be studied. The authors’ conclusion constituted an additional argument for the continuation of research in this direction.

### 2.4. Synthesis of the Literature Regarding Comparisons between Fiber and CO_2_ Laser

In recent years there has been a tendency to replace CO_2_ lasers with fiber lasers. That is why the analysis of literature continued with the identification of works published in recent years, with comparative analyses between the two types of lasers. The objective of this section is to identify the main advantages and disadvantages of each type of laser. Zaitsev et al. simulated CO_2_ and fiber laser cutting for 1.5 and 8 mm thick steel sheets. The authors generated the coefficient and absorbed energy distribution on the cutting surface and found that the absorbed energy intensity distribution was the same for fiber laser and CO_2_ laser. The secondary radiation intensity is low for small thicknesses and must be taken into account at thicknesses greater than 8 mm. They also found overheating of the sidewalls when cutting with the fiber laser [35]. Another comparative analysis between the two types of lasers was performed by Sołtysiak et al. The experiments were carried out on S235JR steel plates with a thickness of 6 mm [36]. For the types of lasers studied, the linear energy calculated by the authors was 55.4 KJ/m. The results showed that a smoother cutting surface was generated using the fiber laser. According to the authors, the disadvantage of fiber lasers is the high purchase price [36]. We also found a comparative analysis of the results obtained by cutting with both types of lasers in Kubišová et al. The results consisted of the evaluation of the surface quality resulting from CO_2_ and fiber laser processing. The authors concluded that the fiber laser provided a better surface quality compared to the CO_2_ laser, but the differences were small [37].

From the synthesis, it can be seen that fiber laser cutting offers better results compared to CO_2_ laser. However, at large material thicknesses, the CO_2_ laser remains a viable option. Furthermore, the purchase price of fiber laser cutting machines is higher compared to the CO_2_ version. The authors consider continuing research in the direction of improving the cutting process of the CO_2_ laser from an energetic point of view. The sizes are necessary for the interpretation of the results obtained in the following. Furthermore, relations 1–18 can also be applied for the fiber laser.

The objectives of the paper consist of the establishment and development of a set of relations for the calculation of the melting efficiency, the application of the relations for the cutting of Hardox 400 steel, the collection of the experimental data, the establishment of the variation ranges for the cutting parameters (speed, pressure, and power), statistical and analytical validation of the proposed relationships, and the establishment of the optimal combination of parameters that ensure maximum melting efficiency. Another contribution consists of establishing a set of relations for the characterization of melting efficiency, which helps to explain the physical phenomena that occur during laser cutting.

The work is structured in sections. The following section contains an overview of the material used and the methods used. The third section describes the research results and discussions related to the results obtained. The fourth section includes research conclusions, limitations, and future research directions.

## 3. Materials and Methods

The choice of input parameters (power, speed, and pressure) was previously justified. The ranking of the input parameters was made based on a full factorial design, which ensured a link with the energy used in laser cutting. The experimental design allowed for statistical and analytical evaluation of the effect of input parameters on melting efficiency. The chemical composition of Hardox 400 steel is shown in Table 2.

The mechanical properties of the steel used are: tensile strength, 1250 N/mm^2^; yield strength, 1000 N/mm^2^; and hardness within the range (370–430) HBV [11,38]. Table 3 contains the parameters used in the CO_2_ laser cutting experiments.

The laser spot interacts with the metal target on a restricted portion where the transfer of laser energy to the material by absorptivity takes place. The energy density of the spot increases under conditions of high laser energy and a small laser beam divergence angle. When the laser light interacts with the material, the incident photons penetrate the metal atoms and a collision occurs between photons and electrons. It results in directed movement of the electron flow under the influence of a variable electric field determined by the Landau relation. According to Maxwell’s theory, around a variable electric field, a magnetic field appears simultaneously that generates an induced circular current. This current is carried through the material under the influence of the variable electric field, producing thermal energy through the Joule effect. Scientific papers show that the absorptivity depends on the laser wavelength, the condition of the steel surface [14], the laser–metal interaction time, and the laser power [32]. Savii presented the Hagen–Rubens law to determine the absorption coefficient [14]:

(19) $$A=112.2\cdot \rho^{\frac{1}{2}},$$

where ρ represents the electrical resistivity. In the case of steel with a resistivity of 4.6 × 10^–7^ Ω∙m, we will obtain an absorption coefficient of A = 0.076.

The heat input from the laser and the Joule effect generates the melt. The thermoconductive and thermocapillary phenomena of the melt contribute to the steel cutting. In the melt, heat is transferred conductively between the layers through the transfer of energy from the molecules of one layer to the other. Temperature gradients are formed that influence the melt viscosity. Draganescu treated the melt in the form of the interaction between the centrifugal force and the weight of the melt [15]. Other authors treated melting in the form of a cylinder rotating between the walls on which the Magnus effect occurs [14]. Khorasani et al. emphasized the Marangoni effect in heat transfer between the melt and the surface of the material [32].

Using CO_2_ laser specific parameters allows setting the power and energy density of the laser beam. The working parameters are the spot diameter, the focal length, and the divergence of the laser beam. The absorption coefficient (A) is used to find the radiation intensity at depth Z. The studied steel is a suitable material for laser processing because it has a low reflection coefficient and implicitly a high absorption. The local working area accumulates heat, which rapidly transforms the heated portion into a pool of molten metal that is subsequently removed by the O_2_ assist gas at a pressure below 1 bar. The role of this state parameter is to maintain the reaction of oxidizing the iron, widening the gap, and pushing the melt toward the bottom edge of the part to penetrate the material [39]. The speed of the laser reduces the input of laser energy, a necessary condition for the flow of molten material to be small and the obtained surface to be smooth. The laser radiation energy for Hardox 400 steel (>1 KJ) can be increased provided the number of photons in the laser tube are amplified to a threshold value.

In the studied steel laser cutting processes, laser power, gas pressure, and cutting speed are the most common input sizes. An experimental investigation with four influencing factors would raise the processing cost and duration of the experiments. Through thermal processing with laser radiation, the variation of the melting process was followed to reduce the consumption of material and energy. Prediction and overall melting efficiency were investigated using statistical models. The role of the prediction is to simulate melting, and the obtained mathematical relationships can develop the laser cutting process through the combined effects of two input parameters (P,p), (P,v), and (p,v).

The central values of the cutting sizes were set by trial tests. Limits of the cutting parameters variation were established for the cases in which cutting is performed (P_min_ = 4900 W, P_max_ = 5100 W, v_min_ = 1700 mm/min, v_max_ = 1900 mm/min, p_min_ = 0.45 bar, p_max_ = 0.55 bar). The selection criterion for the central parameters was the surface roughness. The experiment was organized according to the number and values of the input factors (3 × 3 × 3 = 27).

The cutting mode was CW, the piercing time was set to 0.7 s, the distance between the piece and the nozzle in the piercing phase was 6 mm, the nozzle distance in cutting was 1 mm, the laser power in the piercing phase was 5000 W, the pressure in the piercing was 0.7 bar, the focus position in the piercing was 0 mm, the focus position was +1 mm, and the nozzle positioning height was 40 mm. The auxiliary gas used was O_2_. A nozzle with a conical profile of 1.5 mm diameter was used.

**Figure 1:**
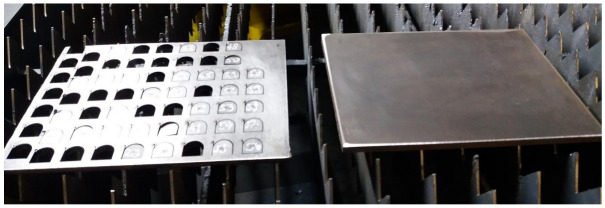
Order of processing.

The semi-finished product in the form of sheets was standardized. The sheets are available in thicknesses between 2 and 30 mm and have as dimensions: width 220 mm, length 300 mm, thickness 8 mm, and mass 5.181 kg. The initial cut plan established is shown in Figure 1. The sheet was carefully analyzed before starting the experiments to verify the provisions of EN 10163-2. The settings made on the laser cutting machine were made so that the cutting experiments were carried out continuously.

**Figure 2:**
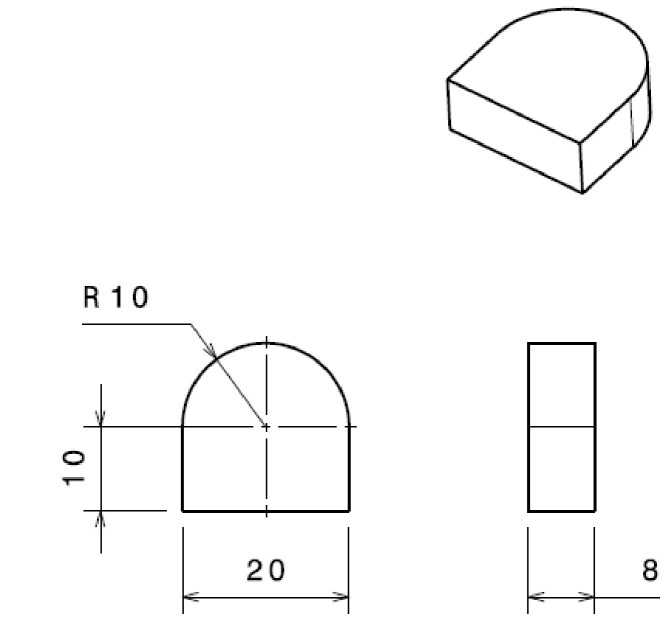
Piece dimensions (mm).

The experimental design consisted of laser machining Hardox 400 steel to obtain a number of 135 parts (27 parts/series ×5 series). Each series consisted of an equal number of experiments. The order of processing the parts is shown in Figure 2. The data obtained were statistically processed with Minitab software, version 19 (State College, PA, USA). The dependent variable was the melting efficiency, and the independent variables were: speed, pressure, and laser power. Two independent parameters are varied and one is held constant to obtain sufficient data on melting efficiency.

A shape of the part containing three straight profiles and one semi-circular was chosen (Figure 2). The piece had the following dimensions: length 20 mm, height 20 mm, and radius of the semi-circular profile 10 mm. Before the experiments started, a reference point was established, and all independent processing was performed. After the initial tests, the benchmark values for the parameters were: speed v = 1800 mm/min, power P = 5000 W, and pressure p = 0.50 bar. Laser radiation emitted continuously by CO_2_ + N_2_ + He gas mixture generators was used for the samples processed from Hardox 400 steel. The melting tool was the laser spot obtained after the lens was crossed by the laser light beam with a diameter of 20 mm. The lens concentrated the light energy into the laser focus with 0.2 mm diameter. The focal spot had a diameter equal to the wavelength laser radiation of 10.6 µm.

A certain amount of heat from the laser spot enters the material, which through thermal convection creates the liquid portion, and by thermal conduction the plate and the piece are heated. The high absorptivity of Hardox 400 steel generates a larger amount of melted material with a given laser energy. Local melting is possible when laser power densities are in the range (10^5^–10^6^) W/cm^2^.

One of the parameters that has important influences on melting is the laser power [40]. For processed parts, it is found that a better melted and cut surface is obtained on the last side with the straight profile. Such a result can be explained by heat diffusion through the piece. It can also be deduced that the melting front is kept constant, which is why the surface obtained is more correct.

When the laser interacts with the metal, the photons penetrate inside and interact with the electrons of the metal that accumulate kinetic energy and instantly transform it into the heat needed for the melting process. Power and speed have influences in defining the melting efficiency of the laser spot. The relationship showing the melting efficiency of the laser spot is:

(20) $$E_{m}\left(\mathrm{spot}\right)=\frac{\pi \cdot v\cdot d^{2}}{4\cdot P},$$

where P is the laser power, v is the laser speed, and d is the spot diameter. In industrial activity, the following relationship will be used:

(21) $$E_{m}\left(\mathrm{spot}\right)=\frac{\pi \cdot v_{\max}\cdot d^{2}}{4\cdot P_{\min}},$$

where maximum speed and minimum power are used.

Experiments carried out on Hardox 400 steel showed the best melting efficiency with the laser spot and the input parameters in Table 4.

The irradiation time is a physical quantity that describes the processing time of the part. In the case of the experiment, the power density of the laser radiation was 3.9 × 10^6^ W/cm^2^, focal length of the lens was 190.5 mm, radiation divergence was 2 mrad, the energy of the emitted laser radiation was 1237.89 KJ. This resulted in a laser pulse duration of 2 ms. The data were obtained under conditions of maximum speed and minimum power. The irradiation time was 3.15 s.

The volume of melted material at penetration is obtained from the law of variation of the laser radiation intensity in the metal:

(22) $${I=I}_{0}\cdot e^{-\mu \cdot z}.$$

The heat flux variation laws:

(23) $${q=q}_{0}\cdot e^{-\frac{x^{2}}{r_{0}^{2}}}.$$

From Equations (22) and (23) result the melted volume:

(24) $$V=\frac{r_{0}^{2}}{\mu }\cdot \left({\mathrm{lnq}}_{0}\;-\;\mathrm{lnq}\right)\cdot \left({\mathrm{lnI}}_{0}\;-\;\mathrm{lnI}\right)=\frac{r_{0}^{2}}{\mu }\cdot (\ln\frac{q_{0}}{q})\cdot (\ln\frac{I_{0}}{I}),$$

where r_0_ is the spot size (mm), µ is the material coefficient (mm^−1^), I_0_ and I are the laser intensity at the entrance and exit of the material, q_0_ and q are the incident and radial heat flow (dQ/dt) in the x and y direction. Substituting I_0_ = 3.6 × 10^6^ W/cm^2^, I = 10^6^ W/cm^2^, q_0/_q = 1000, r_0_ = 0.2 mm, µ = 0.13 results in a volume melted V = 2.61 mm^3^. A circular laser spot emits a Gaussian laser beam that heats a circular region of radius w on the metal target. Starting from the power density of the laser spot with a Gaussian distribution, we can determine with the help of differential and integral calculation by change of variable:

(25) $$e^{-\frac{r^{2}}{w^{2}}}=u,$$

where u varies from 0 to 1, the total power of light irradiating the metal surface.

(26) $${\rho =\rho }_{0}\cdot e^{-\frac{r^{2}}{w^{2}}},$$

(27) $$P_{t}=2\cdot \pi \int_{0}^{\infty }\rho_{0}{.e}^{-\frac{r^{2}}{w^{2}}}\cdot \mathrm{rdr}=\pi \cdot \rho_{0}\cdot w^{2},$$

where ρ_0_ is the power density (W/cm^2^) [15].

## 4. Results and Discussions

Table 5 shows the measured values for the output variable and the melting efficiency in relation to the input variables. Based on the experimental tests in the first series, the melting efficiency value was in the range (14.69–20.68) mm^3^/KJ.

In the cutting experiment for parts 13 and 16, a decrease in melting efficiency was found. This melting efficiency was achieved at minimum speed and medium pressure while the laser power was set to medium or the combination of minimum speed and maximum pressure while the laser power was medium. The experimental block had the lowest melting efficiency of 13.33 mm^3^/KJ at part 25 machined at the minimum speed while the gas pressure and laser power was maximum. It was observed that cutting speed selected at the minimum value contributed to a high laser–material interaction time, and the energy was not fully absorbed by the material, reducing the melting efficiency.

In the case of the cutting experiment, the highest melting efficiency was at a melted volume of 20.68 mm^3^/KJ when 1 KJ of laser energy was consumed, in the case of sample 3, where the power and pressure were minimum while the speed was selected at maximum value. The average melting efficiency obtained from the cutting experiments was 16.35 mm^3^/KJ. Adjusting the cutting parameters at the power of 4900 W with the pressure of 0.45 bar and speed of 1900 mm/min produced an increase in the volume of melted material of 4.33 mm^3^/KJ compared to the average value of melting efficiency.

Small striations located at a distance of 1–2 mm, with a slight deviation from straightness, could be observed on the surfaces of the cut parts. The circular profile was better cut due to the preheating of the part after cutting the straight profile of the part. When the laser entered and exited the contour of the piece, a thermally eroded portion was found due to the accumulation of heat when the speed of the cutting head was reduced and the presence of melting on the lower edge. When the laser exited the material, the nozzle was in the stand-off position. The corners of the piece were straight, but there was a chamfer when the laser changed the cutting direction to 90 degrees. These defects were due to melt flow instability affected by one of the cutting parameters, possibly the cutting speed. There were parts with deeper craters at the exit of the metal laser. Melting was uniform on the cut surface, and instability occurred when the cutting direction is changed. Streaks were more pronounced towards the bottom edge as a result of melt viscosity. A slight tendency to ovalization could be observed in the middle of the surfaces. This is due to the energy received from the laser, the oxidation reaction, and the focus position. When the radiation penetrated the metal, a smooth surface was found due to the combined effect of the input parameters, observations similar to those identified in the papers [41,42].

### 4.1. Melting Efficiency Analysis

**Figure 3:**
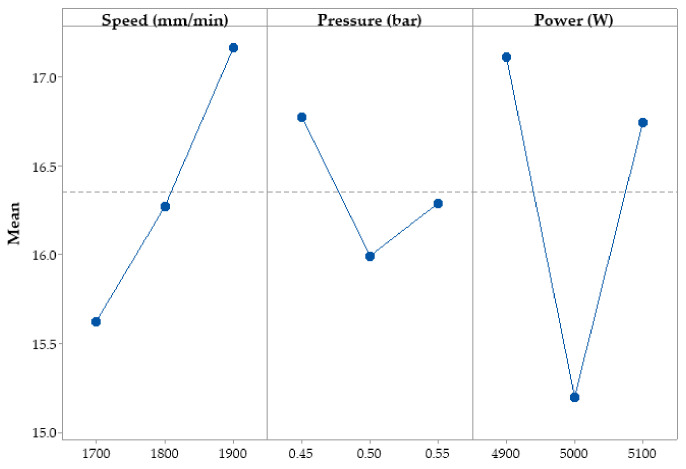
Effect of input parameters on melting efficiency.

The experimental data obtained were statistically processed to establish the influence of the input parameters on the melting efficiency. The influence of input variables on melting efficiency is shown in Figure 3. The main effects plots show the response of the input parameters for each level considered. The *X*-axis contains the input parameter and the *Y*-axis represents the melting efficiency.

It could be observed that an increase in the speed resulted in a growth in the melting efficiency values. The effect was more pronounced in the range (1800–1900) mm/min. Increasing the assist gas pressure between (0.45–0.5) bar had a negative effect on the melting efficiency. At values between (0.5–0.55) bar, the melting efficiency increased slowly. A similar influence was found in the power of the laser. For laser power values between (4900–5000) W, the melting efficiency decreased linearly, and if it increases in the range (5000–5100) W, the melting efficiency increased linearly. The highest efficiency was at minimum power.

The ANOVA method was used to evaluate the influence of the parameters on the response variable. The coefficients R-sq and R-sq. (Adj.) were calculated to establish the influence of the parameters on the melting efficiency. The ANOVA results for the melting efficiency are presented in Table 6. The ANOVA method was applied at a confidence level of 95%. The *p*-value should be less than 0.05 to show that it has a significant influence on the selected answer [43,44].

Table 6 shows that laser power has a moderately significant impact on melting efficiency. Such a result is because the *p*-value for power is close to the 0.05 value. The pressure and speed have no significant influence, p value being 0.196 > 0.05 and 0.636 > 0.05, respectively. The values obtained are R-sq. = 34.61% and for adjusted R-sq., 14.99%. The large difference between the two values shows that there are other predictors. Since *p* value is found to be relatively small 0.049 and Fischer mean F = 3.06, a moderate relationship between melting efficiency and laser power results. The F statistic test shows that there is an average relationship between the response and the predictor. From Table 6, it can be seen that the power input was the highest, 88.53%, followed by speed, 11.43%, and pressure, 0.04%. The reason for the low value of R^2^ is due to large errors that reduce the coefficient of determination. The most sensitive parameter following the ANOVA analysis of variance is the laser power. Independent variables show that they can influence the melting efficiency in a proportion of 34.61%. There are other factors that can influence melting efficiency, such as focus position, nozzle-to-part distance, and material thickness. The adjusted R-sq is small, 14.99%, and has the role of opposing the decrease in R^2^ and penalizing the addition of external factors to the chosen model.

The main effect is due to the power of the laser, which contributes to an energy transfer of the laser spot onto the Hardox plate. Instantaneously, heat is generated locally in the plate by convective transfer determined by Newton’s law and by conductive transfer through the plate governed by Fourier’s law. Each part develops a different melting front due to the variation of laser energy that heats the plate unevenly due to the interaction of predictor factors. In the working area, there is a current density of free electrons that instantly heats the melt, and through the steel plate, the heat wave is due to the vibrations of the ions of the metal network that give rise to the appearance of a heat flow and the movement of electrons. Each part is processed with different melting regimes. The ANOVA method penalizes these processing inaccuracies due to the working parameters. In laser irradiation it is possible to have a variation of the viscosity coefficient of the melt with temperature, which determines a different metal volume. When parts and thicknesses are measured of the steel plate, different values of dimensions are found within the tolerances established by the laser processing technology.

**Figure 4:**
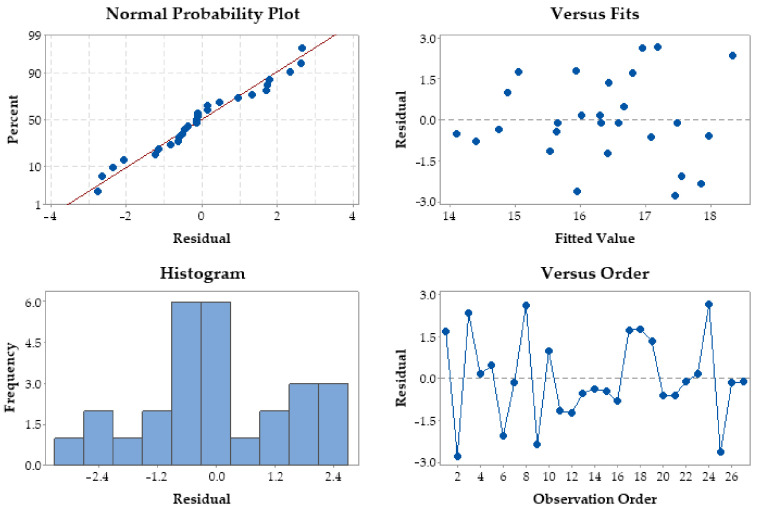
Residual plots for melting efficiency.

Figure 4 shows the residual values for the melting efficiency. When considered together, the residual plot tests show a similar good agreement with melting efficiency, which satisfies the ANOVA results.

A large amount of data with residual error are not in the straight line of the probability distribution, which shows that some of the variability in the data is unexplained [45]. There are some errors that lie on the linear regression line that estimates the distribution of expected errors. The densest residual melt errors are in the ranges (−1.5, 0) and (1.5, 3). The histogram of residual errors contains precise information about the distribution of errors. The maximum error size is 6. The frequency of occurrence of the center class is 6. Most of the data to the left near the mean follow the Gaussian curve, and those to the right do not. The melting efficiency error data to the right of the center class do not follow the normal distribution at class (2, 4) with frequency 3.

The Gaussian plot shows flattening due to the high value of the standard deviation. By residual error we considered the difference between the measured value of the efficiency and the one calculated with the mathematical regression. The cloud of 27 points is very close to the regression line, which shows that the statistical results are correct. The parts are obtained by heating, melting, and sudden cooling. Due to these phenomena, the standard deviation increases, the population of errors is located at a 95% distribution.

**Figure 5:**
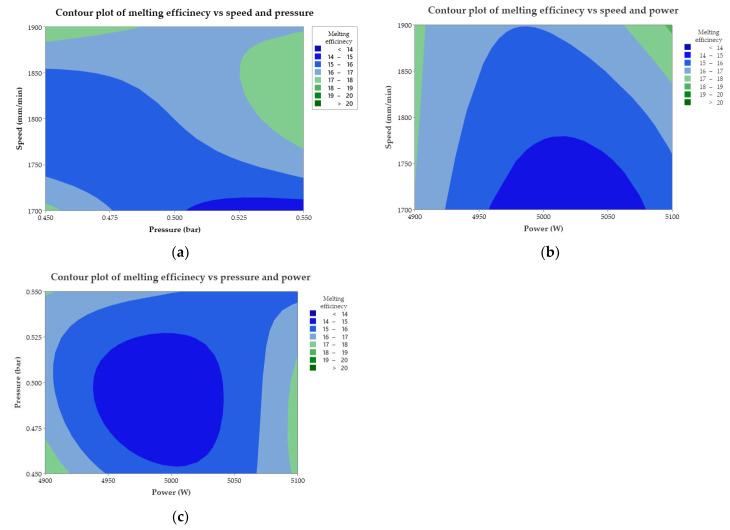
Contour plot for melting efficiency: (**a**) Speed and pressure; (**b**) Speed and power; (**c**) Pressure and power.

The contour plots shown develop the link between efficiency and predictors (Figure 5). They were generated with the contour plot option in the Minitab software, having the role of adjusting and improving the response to the impact of several factors (pressure, speed), (power, speed), and (power, pressure). Through the area and strength of the color, the combination of factors contributing to the increase in melting efficiency, working conditions, and the predictor factor with the greatest influence can be deduced. It follows from the graphs that several parameters act simultaneously on the melting, which is why measures to control the working parameters are required. Consequently, investigation of predictors affecting part melting and cutting is necessary.

At the pressure–speed impact, there is a dispersion of the efficiency shown by the color spectrum. The response prediction indicates that the melting efficiency range of the values is between (17–18) mm^3^/KJ.

The conditions under which these values can be obtained are minimum pressure with maximum speed, minimum pressure and speed, respectively, maximum pressure with speed in the medium–maximum range. At the impact of power with speed, it is found that at maximum power and speed, the melting efficiency has values above 18 mm^3^/KJ. It turns out that the prediction is a simulation model of the efficiency when the laser power density is maximum while the maximum speed reduces the processing time and the additional thermal energy input of the laser radiation to the material. The progressive decrease from these extreme values has the effect of reducing the amount of material melted and ejected. In the combination of power and pressure, it is observed that average values of melting efficiency are obtained under conditions of setting maximum power with minimum–medium pressure or under extreme conditions when power and pressure are minimum. The problem of predicting power with pressure significantly reduces the efficiency through the surface disk (circle or ellipse) with the center at the power and average pressure points, values that will be avoided in production. Contour surfaces for melting, power-speed contour lines are obtained with the Gaussian distribution function. The efficiency power–speed prediction model generates a response of the molten volume within a range of (18–19) mm^3^/KJ. It can be appreciated that the pressure of the assisting gas is not a significant influencing factor because it only influences the intensity of the thermoenergetic reaction and the removal of a limited amount of molten material.

### 4.2. The Linear Predictive Model for Melting Efficiency

**Figure 6:**
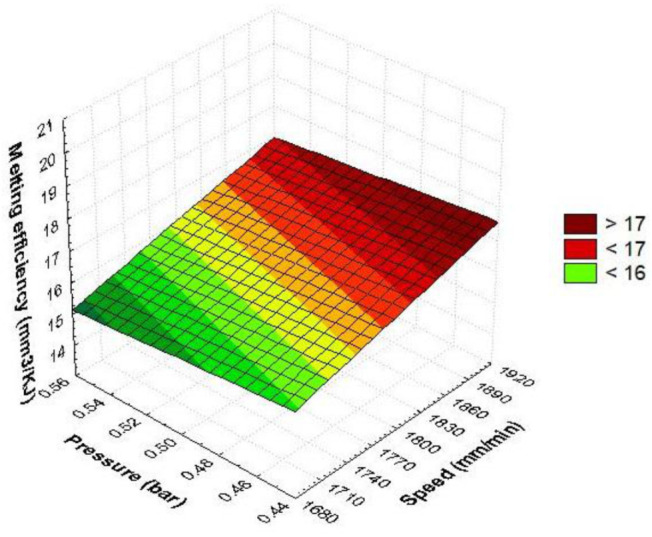
Linear dependence of melting efficiency as a function of pressure and speed.

The linear predictive plot was generated with the response surface method (RSM). It is a 2D surface that includes all combinations of speed and power (Figure 6). Responses vary linearly along a plane surface. This contains different colors that indicate the intensity of the output responses. If the cutting speed is in the range of (1860, 1900) mm/min and the pressure is in the range of (0.44–0.48) bar, the result is a high melting efficiency. Gradually setting the cutting parameters below these values has the effect of reducing the melting efficiency. Maximum values are obtained under conditions of maximum speed and minimum pressure. It is noted that increasing the pressure to the maximum value while maintaining the maximum speed causes a slight reduction in the melting efficiency. Cutting speed has a steeper slope compared to pressure. It follows that speed has a greater influence than pressure.

**Figure 7:**
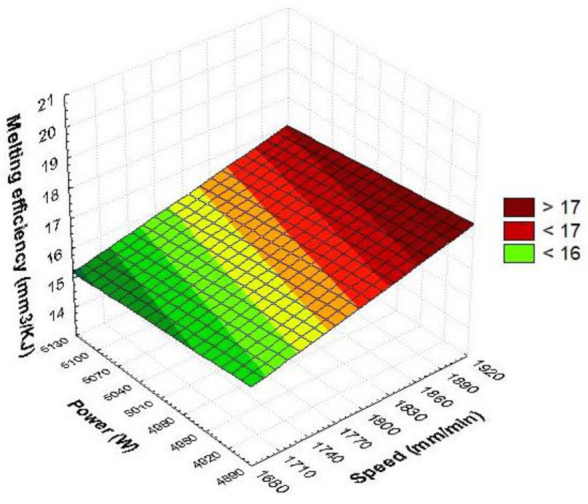
Linear dependence of melting efficiency as a function of speed and power.

Figure 7 contains the interaction between the speed and power results. If the speed is in the range of (1860, 1900) mm/min and the power is in the range of (4900, 4950) W, results maximum melting efficiency. The gradual setting of the cutting parameters from these values has the effect of reducing the melting efficiency.

**Figure 8:**
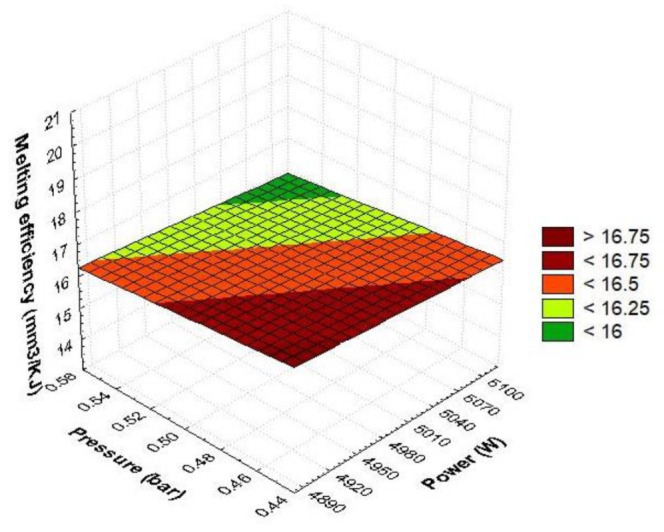
Linear dependence of melting efficiency as a function of pressure and power.

Figure 8 contains the interaction between the pressure and power results. Maximum melting efficiency values are obtained under conditions of maximum speed and minimum power. If the pressure is between (0.45, 0.48) bar and the power has values in the range (4900, 4950) W, a maximum melting efficiency results. Gradually setting the cutting parameters differently from these values has the effect of reducing the melting efficiency. Maximum values are generated under the conditions of minimum pressure and power. It is noted that increasing the pressure to the maximum value causes a slight reduction in the melting efficiency.

Using a regression analysis, relations for each response variable were obtained. The regression equations are presented in the relations (28)–(30).

E_m_ = 4.92 + 0.0077·v − 4.8476·p. (28)

The coefficient of the model is 4.92. The effect induced by increasing the speed is to increase the melting efficiency. Gas pressure is a factor that causes a reduction in melting efficiency.

E_m_ = 11.648 + 0.0077·v − 0.0018·P. (29)

The power acts in the direction of the decrease in melting efficiency, and the speed determines the increase in the output variable. Dominant in this case is the cutting speed, because it has a correlation coefficient of 0.0077, higher than that of the power of 0.0018. The coefficient of the model is 11.648. The speed–power graph increases the speed factor efficiency. The coefficient of the model is 27.9287. Both quantities act to reduce the melting efficiency.

E_m_ = 27.9287 − 0.0018·P − 4.8476·p. (30)

### 4.3. The Quadric Predictive Model of Melting Efficiency

**Figure 9:**
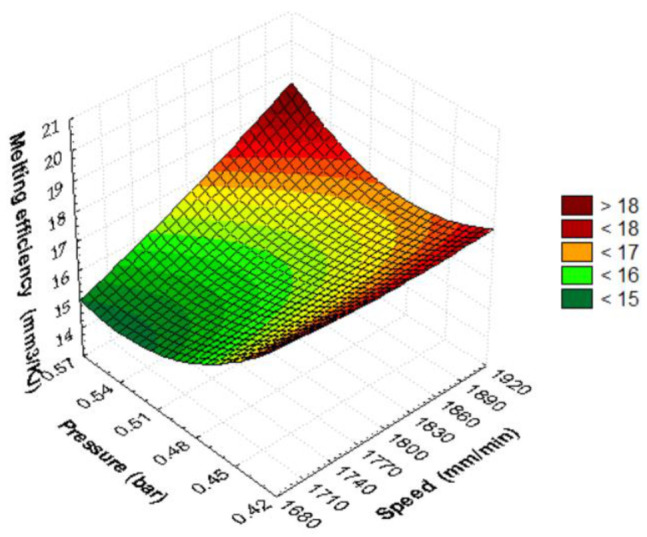
Quadratic dependence of melting efficiency on speed and pressure.

**Figure 10:**
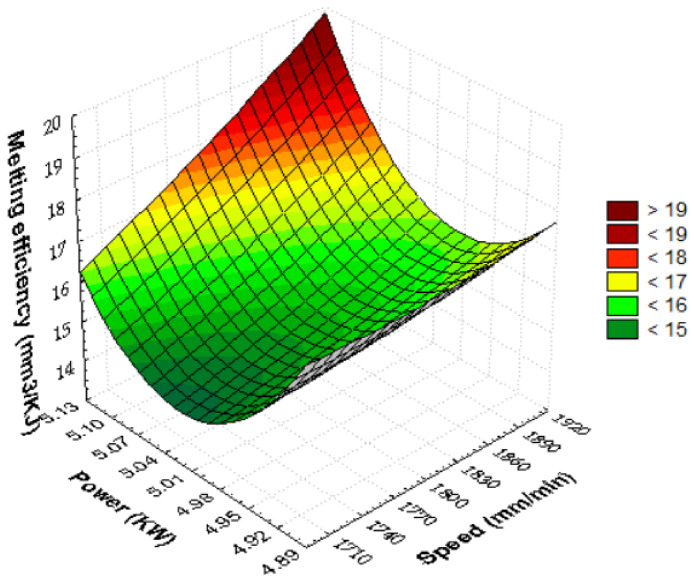
Quadratic dependence of melting efficiency on speed and power.

**Figure 11:**
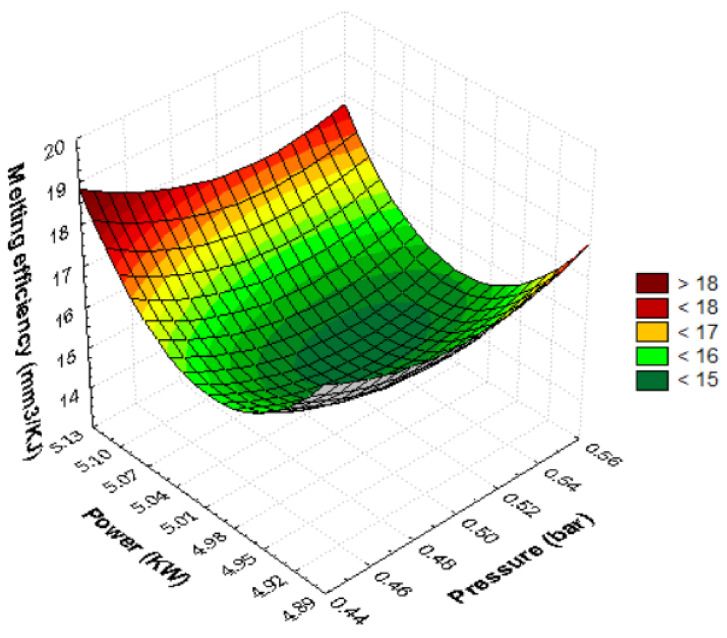
Quadratic pressure–power melting efficiency dependence.

The 3D graph in Figure 9, Figure 10 and Figure 11 provides a conclusive image for anticipating how the considered input parameters affect the melting efficiency. From Figure 9, it is observed that the melting efficiency is maximum when the speed and pressure are at the highest values. As the speed or pressure decreases under maximum setting conditions, the melting efficiency decreases. It reaches its minimum value at minimum speed and maximum assist gas pressure. When the pressure decreases and the speed is kept high, the melting efficiency increases.

The shape of the surface in Figure 9 is quadratic due to the interaction of the parameters at the same time. It is observed that the speed increases the efficiency more rapidly in running conditions towards maximum values. The estimated quadratic model of melting efficiency as a function of pressure and speed using the regression technique is represented in the following relation:

E_m_ = 193.2015 − 0.089·v − 411.7111·p + 1.2111E − 5 v·v + 0.1062·v·p + 215.7778·p·p (31)

In the quadratic relationship, it is found that the linear terms speed and pressure decrease the melting efficiency. Instead, the quadratic interactions and the linear interaction between speed and pressure increase the melting efficiency. The quadric surface presents a maximum melting efficiency under the conditions of a maximum cutting speed, while the gas pressure becomes maximum. The RSM graphical model indicates the most influential parameter is the cutting speed because it rapidly increases the melting efficiency. The shape of the efficiency–pressure graph is moderate, showing that this cutting parameter slowly changes the quadric surface.

In Figure 10, the quadratic dependence of the melting efficiency on the power and speed was represented graphically. The maximum cutting efficiency is obtained when power and speed are maximum, respectively, extreme conditions. Gradually changing the parameters of cutting from this critical point decreases the melting efficiency. The decrease in speed leads to a rapid reduction in the monitored parameter. The shift of power from average values results in an increase in melting efficiency. This means that at extreme values of laser power, an improvement in melting efficiency is found. The estimated quadratic model of melting efficiency as a function of speed and power, using the regression technique, has the following form.

E_m_ = 4905.4144 − 0.3338·v − 1836.8721·P + 1.2111E − 5·v·v + 0.0596·v·P + 172.7778·P·P (32)

The linear and quadratic regression coefficient of power is higher than speed, which indicates that the laser power is significant. If the laser spot delivers maximum laser light energy to the target, it will be attenuated upon entering the material by the cutting speed, which is intended to reduce the interaction time between the material and the laser.

The combination of power and speed set to maximum will result in the largest cut volume from the slot (>19 mm^3^/KJ) at a consumption of 1 KJ of laser energy. The metal absorption capacity is maximum when the power and speed are selected to the maximum value, so that the laser energy is transformed into local thermal energy, which heats and easily melts the largest amount of material. The melting dependence on the speed is quasi-quadratic, and on the laser power, it is quadratic. The efficiency–power graph is more affected than the efficiency–speed graph. It follows that even in this case, the laser power is the main parameter. In industrial production, medium power conditions will be avoided because, in these situations, the quantity of melted and discharged material decreases. It is noticed that the laser power set under different conditions determines three intensity levels, whereas the cutting speed is only two on the square surface. If we select power P = 5100 W while speed v = 1900 mm/min with moderate pressure p = 0.50 bar, we will obtain the highest melting efficiency. For power P = 4900 W and speed v = 1900 mm/min, the melting efficiency calculated is 17.49 mm^3^/KJ.

Figure 11 contains the quadratic dependence of the melting efficiency against pressure and power. The 3D surface obtained has the shape of an inverted paraboloid. Maximum melting efficiency is achieved when the laser power is maximum while the pressure is minimum. Gradually changing the cutting parameters from this critical point decreases the melting efficiency. Moving the power to medium values causes a significant reduction in melting efficiency. The minimum value of the parameter appears at the average power and pressure values. Shifting the pressure towards the minimum or maximum values leads to an increase in the melting efficiency. The estimated quadratic model of melting efficiency, in function of pressure and power, using the regression technique, has the following form:

E_m_ = 4322.3668 − 65.6111·p − 1714.1221·P + 215.7778 p·p − 31·p·P + 172.7778·P·P (33)

The laser melting efficiency is maximum when the power is maximum while the pressure is selected at minimum. The melting response decreases as we move from maximum power to minimum pressure. The amount of molten material in the slot is over 18 mm^3^/KJ when we set the power P = 5100 W with pressure p = 0.45 bar. Substituting into the quadratic polynomial relation, the power = 4900 W and pressure p = 0.45 bar, we will obtain a melting efficiency of 17.37 mm^3^/KJ. The variation shape of the quadratic surface is the same as that of an inverted paraboloid that holds water, which shows minimal melting under controllable parameter setting conditions, pressure with medium power. The dependence of melting on power is more pronounced than the gas pressure factor due to the depth of the parabolas. They are more affected by laser power than by pressure (the contour length of the graph is longer). The mathematical model of regression shows that quadratic factors (power, pressure) raise efficiency, whereas linear pressure–power and interaction terms between them decrease the molten metal content. The increase in efficiency will be achieved under conditions of maximum power and pressure. This combination with two input parameters set to maximum will result in maximum laser power consumption. The gas flow removes a large amount of molten metal, which will increase the intensity of the chemical oxidation reaction and local temperature. It is found that at medium speed, the metal absorbs very well the maximum laser energy, which causes a maximum amount of melted material. The combination of the two predictors (power-pressure) ensures a technological process of manufacturing parts by melting efficient material by setting the maximum power, minimum pressure, and average cutting speed. The extremes of melting efficiency obtained from the RSM plot are at maximum power with minimum pressure above 18 mm^3^/KJ or at medium power and pressure below 15 mm^3^/KJ. Setting the power to maximum (P = 5100 W) with minimum or maximum gas pressure will ensure increased efficiency. Increasing the pressure will have the effect of increasing the assist gas consumption and slowly increasing the melting efficiency. The technological process of melting with minimum laser power presents a longer processing time by decreasing the local accumulation of laser energy, which will generate, through thermal convection, a heat that melts in a longer time interval.

The statistical mathematical method was developed using the response surface method (RSM). The method founded by Box and Wilson in 1951 involves obtaining a response, represented graphically in the form of a surface, based on the behavior of several input variables [43]. The advantages of the method determined its widespread use in the field of laser processing technologies [6,17,20,21,46]. The input data were combined with the responses, resulting in linear and quadratic regression relationships of melting efficiency. After checking the mathematical relationships with the controllable parameter values, the values from Table 7 resulted. The linear mathematical relationship between power and pressure indicates a result closer to the experimental model. Such a result can be used in production activity. If the aim is to increase the melting efficiency, the relationship between the combination of factors, speed, and power will be used. The result is consistent with the cutting efficiency obtained under the conditions of processing at minimum laser power and maximum speed.

Machining experiments are improved from the power–speed adjustment to the average level of the gas pressure. The quadratic model presents a maximum cutting efficiency under the conditions of v = 1900 mm/min, P = 4900 W, and p = 0.50 bar. Compared to the experimental model, the only predictor that differs is the assistant gas pressure. It can be anticipated that high cutting gas pressure increases the intensity of the oxidation reaction; therefore, the amount of melted and ejected material increases. The melting efficiency can be maximized by the quadratic mathematical model because it is more accurate and precise because of the number of terms. Good compatibility is observed between the experimental model (Table 4) and the mathematical model.

## 5. Results Interpretation

### 5.1. The multiple Regression Model

Another objective of the research was the obtaining of new relationships in order to verify and validate mathematical and statistical relationships or other relationships related to the determination of the melted volume with a laser energy consuming 1 KJ. The statistical linear relationships were combined in order to obtain a metal melting efficiency relationship dependent on three influencing factors established in the experiment. The mathematical equation gives us exact information about how melting relates to speed, power, and pressure. The mathematical expression of the quantity studied as an indicator of laser cutting is given by the algebraic relationship:

E_m_ = 14.83 − 0.0051·v − 3.2317·p − 0.0012·P. (34)

The coefficients of multiple regression are: 0.0051 for speed, respectively, 3.2317 for pressure, and 0.0012 for laser power, and 14.83 is the coefficient of the model. The verification of the relationship is carried out by keeping the gas pressure constant, the speed v = 1900 mm/min, and the power P = 4900 W. The values of the melting efficiency are shown in Table 8.

The analysis of relation (34) and Table 8 shows the following:

1. Melting efficiency depends linearly on the influencing factors;
2. Cutting efficiency decreases linearly with gas pressure and laser power;
3. Melting efficiency decreases linearly with cutting speed;
4. The maximum efficiency is 17.19 mm^3^/KJ under conditions of maximum speed and minimum laser power, while the pressure is the lowest;
5. Melting efficiency decreases when the assistant gas pressure increases;
6. The study improves laser cutting by mathematically estimating response efficiency according to predictor factors and multiple regression coefficients. This relationship is in agreement with the results obtained by Seungik and Dongkyoung (2020) et al. using multiple linear regression [47];
7. Multiple regression coefficients decrease from statistically determined linear regression coefficients for all influencing factors when they act simultaneously on melting efficiency;
8. The result is above the average of the experimental model, so it is considered that the mathematical relationship approximates the melting with three interaction factors at the same time quite well.
9. Following the combination of the three quadratic mathematical relationships, we obtained a regression relationship that contains all the influencing factors included in the laser cutting experiment.

E_m_ = 3140.3275 − 0.1409·v − 159.1074·p − 1183.6647·P + 0.8074 × 10^−5^·v^2^ + 143.8519·p^2^ + 115.1852·P^2^ + 0.0354·v·p + 0.0198·v·P − 10.3333·p·P. (35)

For the power 5100 W, the speed 1900 mm/min, and the pressure of 0.45 bar, we obtain the melting efficiency of 16.98 mm^3^/KJ. Regression is found to result in a melting efficiency of <17 mm^3^/KJ. The result of the quadratic efficiency is very close to the linear efficiency, so we can consider that the two mathematical relations containing three influencing factors are compatible to determine the melted volume with a consumption of 1 KJ of laser energy. It is found that the linear terms decrease the melting efficiency, and the quadratic terms increase the efficiency of the processing process. Regression can be considered an alternative to estimate the melting efficiency. The polynomial with more terms can better approximate the metal melting process. Melting errors decrease as interactions between input factors increase. The brief conclusion of this regression research is that the linear model is adequate with the quadratic model. The melting process depends on the input parameters, and no data on the characteristics of the laser light or material properties are required to estimate the volume of metal cut.

### 5.2. Mathematical and Theoretical Model Verification

A verification of the relationship of the statistical regression of melting according to the most significant parameters (laser power and cutting speed) and a comparison of it with the relationship of a Lagrange interpolation model were proposed. This relation of efficiency, dependent at the same time on two important cutting parameters (v,P), was chosen because it most increases the volume of melted material with 1 KJ of energy among all the relations presented in the study. In this relation, the speed was replaced by the maximum value of 1900 mm/min. A quadratic mathematical relationship was obtained that explains the dependence of the melting efficiency according to the laser power:

E_m_ (regression) = 172.7778·P^2^ − 1723.6321·P + 4314.9151. (36)

Since the laser power factor coefficients are high, it follows that this relationship indicates a strong link between melting efficiency and power. The mathematical verification of this relationship is performed by replacing the laser power with the value 5100 W. A high melting efficiency of 18.3419 mm^3^/KJ is obtained, which is consistent with the RSM plot for maximum speed and power. It follows that the relationship between response and power is established by a quadratic polynomial. The statistically determined mathematical speed-to-power relationship is the most appropriate to use in increasing melting efficiency because the laser spot delivers the most laser energy to the part. The most efficient melting experiment is obtained at full power speed while the cutting gas pressure remains constant.

The theoretical Lagrange model was developed based on experimental data and melting responses. It was based on the use of data from the experiment, so that they ensure stability of the melting and cutting experiment. Table 9 shows the input and output data for experiments 1, 14, and 27.

The set of points is x_0_ = 4900 W, x_1_ = 5000 W, x_2_ = 5100 W, and the answers are f(x_0_) = 18.50; f(x_1_) = 14.40, and f(x_2_) = 17.38. The interpolation polynomial is determined from the linear relationship between the function image and the Lagrange basis polynomials:

E_m_ = L(x) = f(x_0_)·l_0_ + f(x_1_)·l_1_ + f(x_2_)·l_2_, (37)

where l_0_, l_1_, l_2_ are the basic Lagrange polynomials developed by numerical analysis as a function of x, x_0_, x_1_, x_2_. The result is the polynomial approximation of the melting efficiency depending on the independent parameter x = P (laser power):

E_m_ (Lagrange) = 0.000354·P^2^ − 3.5456·P + 8892.4. (38)

Using the maximum value of laser power of 5100 W, the melting efficiency equal to 17.38 mm^3^/KJ is obtained. The brief conclusion of this research is that the statistical quadratic model is relatively consistent with the theoretical mathematical model of Lagrange. Efficiency-predictor relationships are important in terms of functionality and melting efficiency values. This approach brings the latest results to laser machining of Hardox steel. The best melting conditions according to statistics are produced when the speed takes the value v = 1900 mm/min, p = 0.50 bar, and P = 4900 W. Table 10 shows the values of the melting efficiency by applying the relations of linear and quadratic regression.

In the linear model, the highest efficiency value is obtained in situations with a laser speed–power impact of 17.45 mm^3^/KJ (Table 11).

In the quadratic model, the highest efficiency value is obtained in the situation with the laser speed–power impact of 17.51 mm^3^/KJ. In the calculations for quadratic efficiency (v,P) and (P,p), the values 4.9 KW and 5.1 KW were used (Table 12).

The theoretical model shows the highest melting efficiency: 18.50 mm^3^/KJ at the minimum laser power (Table 13).

Following the analysis of the models used for efficiency research, we found that the quadratic relationship (speed and power) promises the best melting efficiency greater than 17.5 mm^3^/KJ. The following observations emerge from the graphs obtained with RSM:

- The response variable—melting efficiency was observed using statistical (L) and the quadratic (Q) model. The response surface for slot metal melting analysis by laser machining is a plane or quadric surface;
- On the impact and variation of speed–pressure factors on melting efficiency, the linear plot indicates a maximum efficiency of <18 mm^3^/KJ at a speed of 1900 mm/min with a gas pressure of 0.45 bar, whereas the quadratic plot predicts a maximum efficiency for melting >19 mm^3^/KJ under conditions where the speed with the gas pressure are set to maximum values. The increased pressure causes the rise of molten material from the cut and its evacuation. The cut speed set to maximum reduces the interaction time among the laser spot and the part, ensuring maximum absorption of laser energy;
- At the impact of speed–power influencing factors on melting, a maximum efficiency of <18 mm^3^/KJ can be found from the linear graph under conditions where the speed is 1900 mm/min while the pressure gas becomes 0.45 bar; instead, the quadratic plot shows the maximum melting efficiency >19 mm^3^/KJ at the speed of 1900 mm/min with the maximum power of 5100 W. It is found that in the case of processing at the maximum speed, a high efficiency above 19 mm^3^/KJ is obtained under the conditions in which it is combined with the power or the pressure selected at maximum values;
- At the impact of power–pressure influencing factors, a maximum efficiency of <18 mm^3^/KJ of melting can be found from the linear graph under selected pressure power conditions at minimum values. The power–pressure combination run at low values provides the greatest amount of molten metal under conditions where the speed is maintained constant at the medium level. In this case, the laser energy is supplemented by the energy due to the thermochemical and energetic reaction of iron oxidation, which contribute together to increase the volume of molten metal. The quadratic plot (p,P) shows a melting efficiency >18 mm^3^/KJ when pressure 0.45 bar and power 5100 W. The quadratic interaction between the factors increases the melting efficiency compared to the effect of the linear factor. Therefore, consumption and manufacturing cost rise under operating conditions with at least one parameter set to the maximum value or in combination with two influencing factors running at the maximum level;
- The quadratic model (Q) indicates the best answer regarding how the input parameters should be chosen. The interactions between the factors, each with each other, and the combined interactions between them raise the performance of the melting phenomenon through the comparative analysis of the models (L) and (Q). The quadratic pattern is more suitable for laser melting and cutting processes to be completed in a shorter time with maximum melting efficiency;
- The linear model (L) obtained by RSM provides us with an adjustment and checking of the melting process in the manufacture of industrial products. The quadratic model (Q) is best suited for maximizing the melting efficiency. There are differences in melting efficiency estimates due to the model studied, the restrictions imposed by the statistical software, the remoteness of the response data, and high plots.

Metal melting consists of turning a small portion of metal into a liquid state through an isobaric thermodynamic process until it reaches the melting temperature. The heat required for melting is equal to the molten mass or the latent heat. From a physical point of view, it means that a thermal flux from the laser spot acts on each part, which changes the physical state of the metal through successive phase transformations. Maximizing the volume at the depth of the material and in the direction of the cut is important. Through radial growth, we do not obtain efficiency in melting, but cut material with high energy consumption. By maximum laser power, we mean that we transmit maximum laser energy to the part, which, by coupling with the speed, attenuates the transfer of energy to the parts. In this situation, an optimal energy is obtained that is absorbed by the steel and transformed into heat necessary to melt the material. The assist gas pressure is maintained at the central value as it provides very good conditions for burning and metal removal. Increasing the speed to the maximum value has the effect of reducing the interaction time between the spot and the steel, providing a thermal front with a certain energy to attack the part. This energy produces heat in the metal that, in a very short time, produces melting. Basically, two types of successive physical phenomena are deduced, heating and melting. The best melting working regime can be performed at the maximum speed power, while the pressure is kept constant at the ambient level. The irradiated material can participate in combustion in the presence of oxygen or in a chemical reaction. The melt flow in the material at the piercing is V or A shaped, ensuring the shape of the part. This flow advances through the material, ensuring the cutting process. The interaction of power with speed provides the best energy that the melting flow has in the material, which ensures the increase in the volume of melted material. The fact that the efficiency prediction suggested we choose the power with the maximum speed means that the studied models are suitable to carry out the processing of the parts in these extreme conditions. Our processing conditions ensure minimum energy consumption and a reduction in the manufacturing time of Hardox 400 parts.

All proposed models were applied for series 2, 3, 4, and 5. The results obtained confirm the intervals established for the first series, the differences being insignificant. Future research directions can help to improve melting efficiency. Thus, research can be oriented towards studying the influence of the viscosity coefficient on the melting efficiency, estimating the kinetic energy and the internal energy of the melt that contribute to the laser cutting process, with the analysis of the results obtained on several different steels. Another direction can be to study other factors that can influence the melting efficiency, such as focus position, nozzle-part distance, and material thickness.

## 6. Conclusions

The research consists of establishing the influence of some fundamental parameters on the melting efficiency. Laser power, cutting speed, and assist gas pressure were selected as input variables in the process, while melting efficiency was considered as an output variable. The main conclusions that emerge from the obtained results are:

- The melting efficiency increases progressively as the power and speed increase and decreases under conditions of selecting the laser power at a medium level while the cutting speed is minimum;
- Power and quadratic speed: the linear power–speed interaction has the effect of increasing the melting efficiency while power and speed linearly decrease the analyzed indicator;
- The most important input parameter on the melting efficiency is the laser power, following the ANOVA results. As the power increases, the amount of heat required to melt the steel also increases the volume of material removed;
- There is a very good concordance between the prediction model and the mathematical model of quadratic power ratio (Q), leading to an increase in melting efficiency and an adjustment of input parameters in the processing of Hardox 400 steel.
- The mathematical relationships were validated through case studies where close results of the efficiency established with the statistical model and the theoretical Lagrange model are found, which shows that they confirm each other;
- The mathematical relationships established by the authors can be applied in industry and research to reduce the processing time of parts, energy consumption, and the impact on the natural environment;
- The authors propose an optimization of the melting efficiency under the conditions of the selection of cutting parameters with laser power 5100 W, speed 1900 mm/min, gas pressure 0.50 bar to increase the value of the melting efficiency above 20 mm^3^ /KJ.

## Figures and Tables

**Table 1:** Melt speed and temperature.

Parameter	Viscosity (N∙s/m^2^)	Diameter (mm)	Mass (kg)	Resistance Coefficient (kg∙s)	Specific Heat (J/g∙K)	Speed (m/s)	Temperature (°C)
Value	10^−3^	0.2	1	0.1	0.46	2.093	1460

**Table 2:** Chemical composition Hardox 400 [11].

Alloying Element	C	Si	Mn	P	Cr	Ni	B	Mo
%	0.14	0.69	1.60	0.024	0.3	0.25	0.004	0.25

**Table 3:** Working parameters.

Parameter (Unit of Measure)	Level		
power (W)	4900	5000	5100
pressure (bar)	0.45	0.5	0.55
speed (mm/min)	1700	1800	1900

**Table 4:** Melting efficiency of the laser spot.

Parameters			Melting Efficiency
v = 1700 mm/min	v = 1800 mm/min	v = 1900 mm/min	$E_{m}=12\frac{{\mathrm{mm}}^{3}}{\mathrm{KJ}}$
P = 4900 W	P = 5000 W	P = 5100 W	

**Table 5:** Design of the experiment and measured values of melting efficiency.

Experiment Number	Speed (mm/min)	Pressure (Bar)	Power (W)	Melting Efficiency (mm^3^/KJ)
1.	1700	0.45	4900	18.50
2.	1800	0.45	4900	14.69
3.	1900	0.45	4900	20.68
4.	1700	0.5	4900	16.19
5.	1800	0.5	4900	17.14
6.	1900	0.5	4900	15.51
7.	1700	0.55	4900	16.19
8.	1800	0.55	4900	19.59
9.	1900	0.55	4900	15.51
10.	1700	0,45	5000	15.86
11.	1800	0.45	5000	14.4
12.	1900	0.45	5000	15.2
13.	1700	0.5	5000	13.6
14.	1800	0.5	5000	14.4
15.	1900	0.5	5000	15.2
16.	1700	0.55	5000	13.6
17.	1800	0.55	5000	16.8
18.	1900	0.55	5000	17.73
19.	1700	0.45	5100	17.77
20.	1800	0.45	5100	16.47
21.	1900	0.45	5100	17.38
22.	1700	0.5	5100	15.55
23.	1800	0.5	5100	16.47
24.	1900	0.5	5100	19.86
25.	1700	0.55	5100	13.33
26.	1800	0.55	5100	16.47
27.	1900	0.55	5100	17.38

**Table 6:** ANOVA results for melting efficiency.

Source	DF	SS	MS	F	*p*	Remark
v	2	10.758	5.379	1.77	0.196	Unsignificant
p	2	2.805	1.403	0.46	0.636	Unsignificant
P	2	18.552	9.276	3.06	0.049	Significant
Error	20	60.684	3.034			
Total	26	92.799				

R-Sq. = 34.61%, R-Sq. (Adj.) = 14.99%, S = 1.74189.

**Table 7:** Melting efficiency—comparative results between linear and quadratic models.

Cutting Parameter	Power (W)	Pressure (Bar)	Speed (mm/min)	Melting Efficiency (mm^3^/KJ)	Absolute Deviation (mm^3^/KJ)	Relative Deviation (%)
σ_m_(v,p)-L	4900	0.45	1900	17.37	1.02	6.3
σ_m_(v,P)-L	4900	0.45	1900	17.45	1.1	6.8
σ_m_(P,p)-L	4900	0.45	1900	16.92	0.57	3.4
σ_m_(v,p)-Q	4900	0.45	1900	16.96	0.61	3.7
σ_m_(v,P)-Q	4900	0.50	1900	17.49	1.14	6.9

**Table 8:** Melting efficiency—global average.

Parameter	Speed 1900 (mm/min)		Power 4900 (W)	
Pressure (bar)	0.45	0.50	0.55	Global Average
Melting efficiency (mm^3^/KJ)	17.19	17.03	16.87	17.03

**Table 9:** Input and output data.

Experiment Number	Pressure (Bar)	Speed (mm/min)	Power (W)	Melting Efficiency (mm^3^/KJ)
1	0.45	1700	4900	18.50
14	0.50	1800	5000	14.40
27	0.55	1900	510	17.38

**Table 10:** Verification of the linear relationship for the global average of the melting efficiency.

Power (W)	Pressure (Bar)	Speed (mm/min)	Melting Efficiency (mm^3^/KJ)		
			(v,p)	(v,P)	(P,p)
4900	0.50	1900	17.12	17.45	16.45
5100	0.50	1700	15.58	15.55	16.32

**Table 11:** Melting efficiency values in the linear model.

Power (W)	Pressure (Bar)	Speed (mm/min)	Melting Efficiency (mm^3^/KJ)		
			(V,p)	(V,P)	(P,p)
4900	0.50	1900	16.80	17.51	16.38
5100	0.50	1700	15.26	15.58	16.75

**Table 12:** Dependence of melting efficiency on laser power.

Power (W)	Melting Efficiency–Quadratic (mm^3^/KJ)
4900	18.50
5100	17.38

**Table 13:** Melting efficiency as a function of cutting parameters.

Power (W)	Pressure (Bar)	Speed (mm/min)	Melting Efficiency (v,p,P) (mm^3^/KJ)
4900	0.50	1900	16.46

## Data Availability

The data presented in this study are available on reasonable request from the corresponding author.
